# A decade of managing pediatric major traumatic vascular injuries: insights from a referral center

**DOI:** 10.1007/s00383-024-05887-7

**Published:** 2024-11-13

**Authors:** Norhafiza Ab. Rahman, Dirk von Delft, Alp Numanoglu, Edre Mohammad Aidid, Marion Arnold

**Affiliations:** 1 Department of Paediatric Surgery, Red Cross War Memorial Children’s Hospital, Cape Town, South Africa; 2 Department of Surgery, Kulliyyah (Faculty) of Medicine, International Islamic University Malaysia, Kuantan, Pahang Malaysia; 3 Division of Paediatric Surgery, Department of Surgery, Faculty of Health Sciences, University of Cape Town, Cape Town, South Africa; 4 Department of Community Medicine, Kulliyyah (Faculty) of Medicine, International Islamic University Malaysia, Kuantan, Pahang Malaysia

**Keywords:** Pediatric trauma, Vascular injury, Vascular trauma, Firearms, Anticoagulant

## Abstract

**Purpose:**

Incidence, management, and outcomes of pediatric vascular injuries secondary to non-iatrogenic trauma were reviewed over a decade in our institution.

**Methods:**

A retrospective review of medical records (2013–2022) of major traumatic vascular injuries, focusing on injury profiles, treatment modalities, and clinical outcomes.

**Results:**

Thirty patients with 48 vessel injuries were included. Firearms were the leading mechanism, accounting for 43.3% (*n* = 13) of cases. We identified 29 arterial injuries and 19 venous injuries, with 30 (62.5%) of the overall injuries occurred in the lower extremities. Shock (17; 56.7%) and associated injuries (25; 83.3%) were common. Surgery was the most common management strategy. Autologous bypass graft was the most frequently performed procedure for arterial injuries (8; 42.1%), while ligation dominated in venous injuries (9; 64.3%). Blood transfusion requirements (24; 82.7%) and post-operative prescription of anticoagulant and antiplatelet agents (12; 41.4%) were similar for arterial and venous injuries (*p* > 0.05). Three patients demised, resulting in a 90% survival rate. Neither the mechanism of injury, anatomical location, and presence of shock on arrival nor the baseline hemoglobin level served as predictors of mortality.

**Conclusion:**

Intensive resuscitation with blood transfusion and prompt surgical intervention achieve favorable survival rates for pediatric traumatic vascular injuries. Optimal post-operative anticoagulant and antiplatelet regimens remain unclear.

**Supplementary Information:**

The online version contains supplementary material available at 10.1007/s00383-024-05887-7.

## Introduction

Significant non-iatrogenic vascular injuries pose several challenges for pediatric surgeons. They demand rapid diagnosis and management, but identifying these injuries can be difficult as vasospasm and co-existing injuries may obscure the clinical picture. Furthermore, the typical hard signs of vascular injuries are not readily visible in young children [1].

Best acute and long-term management are also unclear in the pediatric population. Decision on management often relies on the patient’s overall condition involving presence of shock and the severity of other associated injuries. Surgical repair presents a challenge due to the small diameter of vessels in children and the necessity of vascular patency for sufficient growth. Other ways of management include expectant or non-operative management and endovascular, both of which have limited data in this group of pediatric patients.

After the acute management, another challenge is the implementation of anticoagulation or antiplatelet therapy. Various studies describe a diverse range of therapeutic approaches and a standardized protocol for pediatric traumatic vascular injuries has yet to be established [2, 3]. Much of the current practice is adapted from that in adults, who have a higher risk for endothelial dysfunction and resultant vessel thrombosis, as well as the information obtained through experience with pediatric organ transplants [4, 5].

This study aims to assess the management strategies used for pediatric traumatic vascular injuries involving limbs, torso, and neck over a decade in our institution. Additionally, the other study objectives include examining injury profiles, analyzing the blood product requirements and post-operative anticoagulation therapies and evaluating the short-term outcomes following the injuries. In conclusion, this effort aims to contribute to the understanding and improvement of peri-operative care in the context of major pediatric traumatic vascular injuries.

## Methods

A retrospective review was conducted for patients who presented to our institution with major vascular injuries between January 2013 and December 2022. The Red Cross War Memorial Children’s Hospital (RCWMCH) is one of the major referral centers for trauma in the Western Cape Province of South Africa for children under 13 years of age, our institutional and provincial upper age limit for pediatric care. Major vascular injury was defined as injury to a vessel of large caliber with an end-organ supply or supplying a large part of a body region which required intervention following damage to achieve re-vascularization or hemostasis. Hypovolemic shock was defined by the presence of persistent tachycardia with hypotension, or tachycardia without hypotension but with signs of impaired end-organ perfusion including raised serum lactate and altered mental status, supported by clinical findings of low pulse volume and tachypnoea, necessitating fluid resuscitation. Tachycardia and hypotension were defined according to the age-specific normal thresholds based on established pediatric reference ranges. We included vascular injuries involving limbs, torso, and neck due to trauma. We excluded patients who succumbed before definitive management, cerebrovascular trauma, cases of which their primary injury treatment occurred somewhere else before transfer and if the cause of injury is iatrogenic.

Patients were identified from the operative database maintained by the Department of Pediatric Surgery and the trauma admission database to the Pediatric Intensive and Critical Unit (PICU) of RCWMCH. The author then reviewed the individual charts of the identified patients. Data on demographic information, injury characteristics, management strategies employed, and clinical outcomes were extracted and analyzed. Simultaneously, overall data on trauma admissions were obtained from trauma and injury database provided by the ChildSafe Research and Educational Centre of RCWMCH. Approvals from our institutional review board and ethical committee were obtained (HREC REF 243/2023 and RCWMCH: RCC 372/WC_2023_04_032).

Descriptive data analyses were performed using IBM SPSS Statistics version 29. Numerical data were described by the median and categorical data were described by frequencies and percentages. Univariate analyses to measure associations between the variables were conducted based on type of data, data normality, and other statistical assumptions as necessary. Statistical significance was set at *p* < 0.05.

## Results

### Patient characteristics

Over the period of 10 years, from January 2013 to December 2022, a total of 64,077 patients presented to the RCWMCH trauma unit, of whom 16,627 (25.9%) were then admitted for further treatment. 48 patients (0.07%) passed away upon arrival. Within the cohort of admitted patients, 30 children had major vascular injuries affecting a total of 48 vessels, making up 0.2% of the overall admissions. The characteristics of patients with vascular injuries are summarized and compared in Table 1. Males (21; 77.8%) predominated, with a higher incidence of male gender compared to the general trauma unit attendees [where at least 37,670 (58.8%) were males with data on gender missing for 3.4%]. Median age of presentation was 9 years and 2 months.

**Table 1:** Patient characteristics (patients, *n* = 30)

		Total	Survived until discharge					
			Yes (*n* = 27)			No (*n* = 3)		
			*n*	%	Median (IQR)	*n*	%	Median (IQR)
Gender	Male	24	21	77.8		3	100.0	
	Female	6	6	22.2		0	0.0	
Age (in months)		110 (94)			107 (92)			141 (–)
Weight (in kg)		30.0 (22.5)			30.0 (22)			30.0 (20)
Mechanism	Firearm	13	12	44.4		1	33.3	
	Sharp object	4	4	14.8		0	0.0	
	Dog bite	1	1	3.7		0	0.0	
	Road traffic accident	11	9	33.3		2	66.7	
	Fall from height	1	1	3.7		0	0.0	
Hemoglobin on arrival (in g/dl)		8.6 (4.0)			8.8 (3.3)			5.7 (–)
Presence of shock on arrival	Yes	17	14	51.9		3	100.0	
	No	13	13	48.1		0	0.0	
Associated injuries	Yes	25	22	81.5		3	100.0	
	No	5	5	18.5		0	0.0	
Associated injuries, yes^a^	Head injury	3	3	13.6		0	0.0	
	Hemo/pneumothorax	3	3	13.6		0	0.0	
	Abdominal solid organ injury	5	5	22.7		0	0.0	
	Abdominal hollow organ injury	4	3	13.6		1	33.3	
	Long bone fracture	13	11	50.0		2	66.7	
	Pelvic fracture	3	2	9.1		1	33.3	
	Others	15	13	59.1		2	66.7	
Management of associated injuries	No	3	3	11.1		0	0.0	
	Yes	22	19	70.4		3	100.0	
	Not applicable	5	5	18.5		0	0.0	
Management of associated injuries, yes^b^	Liver packing/repair	2	2	10.5		0	0.0	
	Splenectomy	0	0	0.0		0	0.0	
	Stoma creation/clip and drop	1	1	5.3		0	0.0	
	Stomach/bowel anastomosis	2	2	10.5		0	0.0	
	Relook laparotomy	3	3	15.8		0	0.0	
	Fixation	9	8	42.1		1	33.3	
	Wound debridement	9	8	42.1		1	33.3	
	Others	9	8	42.1		1	33.3	
Time to intervention (in hour) from presentation^c^	3 or less	12	10	41.7		2	66.7	
	4 to 6	5	4	16.7		1	33.3	
	7 to 9	3	3	12.5		0	0.0	
	10 to 12	1	1	4.2		0	0.0	
	More than 12	6	6	25.0		0	0.0	

Values are presented as number (*n*), median (interquartile range, IQR), or percentage (%)

^a^One patient might have more than one associated injury

^b^Total is not 100% as not all patients with associated injuries had management (of the associated injuries) and one patient might have had more than one procedure done

^c^Total is not 100% as not all patients had intervention (for the injuries)

There was a wide range of causative mechanisms. Penetrating injuries (18; 60%) were caused by firearms, sharp objects, and dog bite in one. Blunt injuries (12; 40%) were mostly caused by road traffic accidents (RTA) and a fall from a height in one patient. More than half of the patients (17; 56.7%) presented with shock, suffered from other associated injuries (25; 83.3%), and subsequently had management of the associated injuries alongside intervention for the injured vessels. Surgical intervention was indicated in patients who presented with hard signs of vascular injury (e.g., active hemorrhage, limb ischemia), hemorrhagic shock, or other life-threatening condition such as peritonitis. Most of the patients (12; 40%) received intervention within 3 h of presentation.

### Vascular injuries by location

Table 2 summarizes the anatomical location of injured vessels. Arterial injuries dominated, accounting for 60.4% (*n* = 29) of the total, while venous injuries constituted the remaining 39.6% (*n* = 19). Femoral and popliteal arteries were the most common vessels injured. Most of the arterial injuries occurred in the lower extremities, followed by the torso, neck and upper extremities. A similar pattern was observed in venous injuries, but with no upper extremity injuries. Eight patients (26.7%) presented with concomitant arterial and venous injuries of the same named anatomic vessels over the extremities.

**Table 2:** Vascular injuries by location (vessels injured, *n* = 48)

Parameter		Anatomical locations of arterial injury				
		Neck	Torso	Upper extremities	Lower extremities	Total
Vessel(s) involved (artery)	Axillary artery	0	0	1	0	1
	Brachial artery	0	0	1	0	1
	Carotid artery (branch)	1	0	0	0	1
	Common carotid artery	1	0	0	0	1
	Common iliac artery	0	1	0	0	1
	External carotid artery	1	0	0	0	1
	Femoral artery	0	0	0	9	9
	Hepatic artery	0	1	0	0	1
	Internal iliac artery	0	1	0	0	1
	Popliteal artery	0	0	0	9	9
	Renal artery (branch)	0	1	0	0	1
	Tibial artery	0	0	0	2	2
	Total	3	4	2	20	29

Parameter		Anatomical locations of venous injury				
		Neck	Torso	Upper extremities	Lower extremities	Total
Vessel(s) involved (vein)	Brachiocephalic vein	0	1	0	0	1
	External iliac vein	0	1	0	0	1
	External jugular vein	1	0	0	0	1
	Femoral vein	0	0	0	5	5
	Great saphenous vein	0	0	0	1	1
	Inferior mesenteric vein	0	1	0	0	1
	Inferior vena cava	0	3	0	0	3
	Internal iliac vein	0	1	0	0	1
	Internal jugular vein	1	0	0	0	1
	Popliteal vein	0	0	0	3	3
	Tibial vein	0	0	0	1	1
	Total	2	7	0	10	19

### Management strategies

Most arterial and venous injuries underwent surgical management, which includes both vascular surgery and primary limb amputation (Table 3). Vascular surgical approach differed significantly between venous and arterial injuries. Excluding five patients who had mangled limbs, autologous saphenous vein bypass graft was the most performed procedure for arterial injuries (*n* = 8, 42.1%), followed by ligation (*n* = 4, 21.1%), primary repair (*n* = 2, 10.5%), and patch repair (*n* = 2, 10.5%). Additional procedures performed were removal of an avulsed bone segment compressing an artery following an arterial repair and ligation for hemostasis with wound dressing for a mangled limb with arterial compromise while awaiting consent for amputation. Ligation dominated in venous injuries (*n* = 9, 64.3%). Where repair was attempted for venous injuries, primary repair was most performed (*n* = 3, 21.4%) followed by patch repair and autologous saphenous vein bypass graft (*n* = 1 each; 7.1%). Fasciotomies were performed when there was concern for compartment syndrome following extremity injuries in 20.7% (*n* = 6) of arterial injuries and 15.8% (*n* = 3) of venous injuries, all for lower limbs in our series. Fasciotomies were performed prophylactically, but not routinely, in cases of ischemic time greater than 6 h, especially with combined arterial and venous injuries, in patients with tense lower limb compartments following vascular repair, at the discretion of the operative team. Emergency laparotomy followed by leaving a laparostomy or open abdomen due to concern of abdominal compartment syndrome was also performed in some patients, due to associated abdominal injuries in these cases.

**Table 3:** Management strategies of vascular injuries (vessel injured, *n* = 48)

Parameter		Vessel type				*p* value
		Artery (*n* = 29)		Vein (*n* = 19)		
		*n*	%	*n*	%	
Management	Expectant	4	13.8	1	5.3	0.78
	Endovascular	1	3.4	0	0.0	
	Surgical	24	82.8	18	94.7	
Type of vascular surgery (if applicable)^a^	Primary repair	2	10.5	3	21.4	0.02*
	Patch repair	2	10.5	1	7.1	
	Bypass, graft—autologous	8	42.1	1	7.1	
	Ligation	4	21.1	9	64.3	
	Others	3	15.8	0	0.0	
Adjunctive procedure (if applicable)^b^	Fasciotomy (all lower limbs)	6	42.8	3	23.1	0.35
	Laparostomy	3	21.4	6	46.1	
	Primary amputation (all lower limbs)	5	35.8	4	30.8	

Values are presented as number (*n*) or percentage (%)

*Significant at *p* < 0.05

^a^Total is not 100% as not all patients had vascular surgery

^b^Total is not 100% as not all patients had adjunctive procedure

Table 4 summarizes the medical management regarding overall peri-operative blood transfusion requirement and post-operative anticoagulant and antiplatelet prescriptions. 82.8% of arterial injuries and 89.5% of venous injuries required blood transfusion. The median volume of packed cells transfused was 35 ml/kg for arterial injuries and 40 ml/kg for venous injuries that spread over a period of several hours to days, corresponding to 1050 ml (three adult units) and 1200 ml (3.4 adult units), respectively, based on the median weight of the patients. Six patients were transfused with more than 40 ml/kg of packed cells acutely, including two of the mortalities, which met the definition of massive blood transfusion [6]. The ratio of packed cells to platelets and fresh-frozen plasma units transfused was 3:2:2.4 for arterial injuries and 3.4:1.8:2.4 for venous injuries; all differences were not statistically significant.

**Table 4:** Management strategies of vascular injuries (continued) (vessel injured, *n* = 48)

Parameter		Type						*p* value
		Artery (*n* = 29)			Vein (*n* = 19)			
		Median	*n*	%	Median	*n*	%	
Blood transfusion								
Yes			24	82.8		17	89.5	0.687
No			5	17.2		2	10.5	
Blood transfusion amount according to type (ml/kg)								
Packed cell		35			40			0.52
Platelet		20			18			0.24
FFP		20			20			0.61
Cryoprecipitate		20			20			0.46
Anticoagulant/antiplatelet use								
Yes			12	41.4		3	15.8	0.11
No			17	58.6		16	84.2	
Anticoagulant/antiplatelet type^a^								
Unfractionated heparin	Yes		8	28.6		1	20	0.78
LMWH	Yes		12	42.8		3	60	
Aspirin	Yes		8	28.6		1	20	

Values are presented as number (*n*) or percentage (%)

*Significant at *p* < 0.05

^a^One patient might have more than one medication administered

Anticoagulant and antiplatelet therapies were administered in 41.8% of arterial and 15.8% of venous injuries, with low-molecular-weight heparin (LMWH) being the most popular option. Eight patients were started on unfractionated heparin before switched to LMWH in seven. Five patients were started with LMWH from the beginning and no patient was started with aspirin (acetylsalicylic acid) as the only agent. Ten patients with arterial injuries received LMWH, including one patient with a primary repair, as well as two patients with isolated venous injuries [great saphenous vein graft repair and inferior vena cava (IVC) bovine pericardial patch repair]. Total duration of prophylactic LMWH ranged from 3 to 36 days, with a median duration of 10 days (interquartile range 6.5 to 14 days) excluding one patient with an IVC injury repaired with a bovine pericardial patch who had 10 months of LMWH for a partially occlusive deep venous thrombosis. On discharge, seven patients (with eight arterial and one venous injuries) received aspirin, while five other patients (with three arterial and two venous injuries) were discharged with LMWH. Aspirin was continued for a duration of 1 month (in six patients) to 3 months (in one patient). No anticoagulation or antiplatelet therapy was given in three patients, all with primary venous repairs (two with IVC repairs and one with an internal jugular repair).

### Outcomes

Survival rate to hospital discharge was 90%, with a median length of PICU stay of 4 days and a median total hospital length of stay (LOS) of 11 days (Table 5). Half of the patients required further intervention following the initial vascular intervention, which included wound debridement, re-look laparotomy, and wound closure. Only one patient from this group required vascular surgery revision as the initial autologous graft repair for his arterial injury was occluded by the pin that was inserted for his femur fracture. Overall, following primary/patch/graft repair, there were 15 patients with 16 preserved vessels. Six had early post-operative imaging within 1 week of repair and one patient with a bovine pericardial patch repair to the IVC developed a partially occlusive chronic deep venous thrombosis which resolved after follow up of  10 months. Unfortunately, post-discharge follow-up data for some patients were unavailable as some were followed up by vascular surgeons at another hospital, while others were managed at their base hospitals. Follow-up data from the study center were available for eight patients with nine vessel injuries. They were followed up both clinically and with duplex or Doppler ultrasound confirming patency in five patients at least a month after repair, excluding the patient with the IVC bovine patch repair described above.

**Table 5:** Short-term outcomes (patients, *n* = 30)

Parameter		*n*	%	Median
Survived until discharge	Yes	27	90.0	
	No	3	10.0	
Length of PICU stay (in days)				4
Length of hospital stay (in days)				11
Need for re-intervention (after definitive intervention)	Yes	15	50	
	No	15	50	

Values are presented as number (*n*) or percentage (%)

Three cases of patients who demised were analyzed.

*Patient 2*: A male aged 11 years and 9 months suffered severe pelvic and lower limb injuries, including vascular trauma to multiple vessels following an RTA. His hemoglobin on admission was 3 g/dl and he was in shock. He had injuries to the common iliac artery, internal iliac artery, femoral artery, internal iliac vein, and external iliac vein. Despite aggressive medical intervention, including transfusions and hindquarter amputation, he succumbed to polytrauma with multiorgan failure by 12 h of admission.

*Patient 6*: A 12-year-old male was involved in an RTA. He sustained traumatic below-knee crush injury and traumatic amputation. He presented in shock with a hemoglobin level of 5.7 g/dl. His femoral artery and vein were severely injured, hence ligated. He experienced cardiac arrest due to hyperkalemia and acute renal failure, leading to his demise after 96 h of presentation.

*Patient 17*: A 2-year-old male with a firearm injury was brought in shock despite an initial hemoglobin level of 10 g/dl. He sustained femur fracture, thigh and flank soft-tissue injury, as well as femoral artery and vein injuries. He underwent primary repair of the femoral artery and femoral vein ligation. He had cardiac arrest twice, once in the emergency unit and again in the operating theater following his surgery, ultimately resulting in his demise around 8 h after presentation.

### Risk factors for mortality

There was no significant difference in terms of patient age, weight, hemoglobin level on arrival, gender, injury mechanism, presence of shock upon arrival, management strategies, and intervention timing between survivors and those who died (refer Supplementary Tables 1.1 and 1.2). Supplementary Table 2 presents an analysis of the association between mortality and location of injuries. However, no significant association was found.

## Discussion

### Presentation

Major vascular injuries secondary to non-iatrogenic trauma account for only 0.2% of all trauma admissions from our decade-long study and are known to be rare in children [7]. Boys dominated our cases at 78%, a pattern that is consistent across different studies [7–11]. In delineating the mechanism of trauma causing major vascular injuries in children, a crucial distinction arises between penetrating injuries and blunt trauma. Penetrating injuries were the most common cause, with 43.3% cases caused by firearms, one of which resulted in fatality. This was followed by 36.7% due to blunt injuries caused by RTA, which contributed to the other two fatalities. Blunt trauma often manifest with a more complicated multisystem involvement, adding challenge to vascular injury diagnosis and management [7]. The pattern of injury mechanisms exhibits variability across the globe [7, 9–11]. Injuries caused by firearms can result from assault, self-inflicted incidents or unintentional events. In our series, all cases were due to assaults, although in some cases, the children were not necessarily the primary targets [12].

Seventeen of our patients (56.7%) presented with shock and associated injuries were present in 25 (83.3%), a number similar in a Canadian study by Kayssi et al. [10]. Therefore, as expected, blood transfusions were required in 82.8% of arterial injuries and 89.5% of venous injuries, with no difference in the blood product used between the groups. The same study by Kayssi et al. involving 106 patients reported 82% of patients had concomitant injuries, with orthopedic injuries being the most common [10]. Similarly, long bone fracture was the most common associated injuries in our series.

We observed a predominance of arterial injuries over venous injuries, with the lower limbs being the most common anatomical locations of injury from both groups. Arteries were consistently reported as the most frequently injured vessels across several studies [7, 10, 13]. Clinically, arterial injuries were easier to diagnose due to their prominence, whereas venous injuries, although potentially more common, may have gone undiagnosed. Our higher incidence of lower limbs vascular injuries for both arteries and veins along with a low rate of upper limb vascular injury (two arteries and no vein) contradict the previous reports by Perea et al. and Wahlgren et al. [7, 9]. The high incidence of extremity vascular injuries provides an opportunity to educate first responders or bystanders about the usage of tourniquets to stop bleeding, potentially saving lives.

### Management

Surgery predominated management strategies for both arterial and venous injuries in our series, but the choice of surgical management differed between the two groups. Autologous bypass graft was the most frequently performed procedure for arterial injuries, while ligation dominated in venous injuries. Definitive vascular repairs were performed in 78.9% of arterial injuries and 36.7% of venous injuries. None of our cases involved the use of a damage-control temporary vascular shunt, which may be considered in patients in shock [14].

Open surgery is the most common management strategy for pediatric vascular injuries, with different studies reporting its usage in up to 80–100% of their series [8, 11, 13]. Perea et al. in one of the largest series reported open repair in 75% of their cases, non-operative management in 21.7% and an endovascular approach in 3.3% [7]. We only recorded a single case that had endovascular management in a patient with injury to a branch of his hepatic artery. This pattern is similar across different studies, highlighting the limited use of endovascular approach in children [8, 9, 11]. Only six vessels in our series were managed non-operatively. This approach was typically reserved for hemodynamically stable patients who did not demonstrate any obvious hard signs of vascular trauma [10]. The primary aim of surgically managing any vascular injury is to secure hemostasis while re-establishing vascular flow as much as possible. A total of five arterial injuries and four venous injuries had primary amputation performed as part of their damage-control surgery. Our data differ from the previous reports. Primary vascular repair was performed in 49% of cases in a Canadian series [10]. Lyons et al. who reported patients with lower extremity arterial injuries found that both autologous saphenous vein graft and synthetic graft predominated at 36%, respectively [1]. A nationwide study involving 34 centers in Sweden reported that primary repair was performed in 12% of cases, interposition grafting in 24%, patch repair in 19%, and bypass in 9.5% [9]. The use of synthetic grafts in children is hampered by size mismatch and concerns for future growth, leading to preference for autologous tissue, with the saphenous vein usually harvested from the contralateral limb.

### Outcomes

Great care must be taken to preserve vessel patency after repair. Strategies to achieve this include meticulous surgical repair and the intra-operative administration of anticoagulant and antiplatelet therapy, another area with limited information in children. Anticoagulant or antiplatelet is recommended for patients considered at high risk of vessel occlusion or when venous trauma is associated with high risk of venous thrombosis [13]. Franchin et al. following their review of pediatric patients, stated that in cases of major arterial injuries repaired with grafts, whether autologous or biological, consideration to the usage of antithrombotic agents should be given to maximize conduit patency [15]. Kirkilas et al. in another study of arterial injuries in children reported the usage of various forms of anticoagulant or antiplatelet agents in 39% of their patients and administered for various durations [3]. Shahi et al. described their experience with their pediatric patients with vascular injuries, except those with contraindications or those who had vessel ligation [2]. Post-operatively, they recommend using heparin for major arterial injuries and transitioning to aspirin upon discharge. For venous injuries, they recommend using enoxaparin, an LMWH, which is continued until discharge. The duration of treatment depends on the type and size of the vessel, the type of repair, and the overall experience of the surgeon. They also suggest consulting a vascular surgeon for more complex injuries. This paper by Shahi et al. represents the most convincing recommendation for post-operative anticoagulation and antiplatelet in children thus far. Our data revealed the highest usage of LMWH in arterial injuries, followed by both unfractionated heparin and aspirin. The higher number of medications prescribed in arterial injuries compared to venous injuries in our series was because more arteries were left in situ after repair, rather than veins that were ligated. Long-term vascular insufficiency causing growth deficits with leg-length discrepancy was not assessed in our population.

Our survival rate was 90% despite half of the patients having required re-interventions after the initial definitive management. Encouragingly, this mortality rate of 10% is comparable with other published studies. Perea et al. reported a mortality rate of 15.3%, with all fatalities attributed to injuries over torso [7]. Another study by Eslami et al. reported a mortality rate of 7.9% [11]. However, it is important to note that the overall reported mortalities may underestimate the true impact of vascular injuries as some patients succumb before the vascular injury is recognized. Perea et al. observed a mean ICU stay of 5.4 days and a mean hospital LOS of 12.5 days, consistent with our numbers of 4 and 11 days, respectively.

All three cases of mortality in our series presented with shock and suffered from severe vascular injuries to multiple proximal lower limb vessels and pelvis in one of the patients. Early profuse bleeding is the main cause of mortality; therefore, the more proximal the vessel is, the higher the mortality risk. All three of our mortalities in our series involved femoral artery injuries. Injuries to the common femoral artery injuries were found to be significantly associated with a higher rate of in-hospital mortality in another report [8]. The same study by Prieto et al. also reported that popliteal and anterior tibial artery injuries were associated with a higher risk of amputation. Our analysis of our results, however, revealed no significant risk factors for mortality compared to survivors. Information on response to initial resuscitation was not completely available in this retrospective study. A focus on hemorrhage control with direct pressure, damage-control procedures with ligation or stenting of vessels instead of repair and correction of coagulopathy and acidosis, is more appropriate in patients with persistent cardiovascular instability.

### Limitations

This study has several limitations. First, its retrospective design limits the accuracy and availability of the data collected. Additionally, our dependability on largely paper-based medical records for data collection may have resulted in incomplete or missing data. Furthermore, due to the rarity of the condition, our study is limited by the small sample size, despite being conducted in a major referral center. Moreover, the study was conducted at a single center, which may limit the generalizability of our results.

## Conclusion

In conclusion, our study found a favorable outcome for the management of most major traumatic vascular injuries, despite most patients presenting with complicated injuries. This highlights the importance of rapid diagnosis and intervention, along with intravenous fluid and blood product administration. Prescription of anticoagulant therapies for those with preserved vessels was common practice. Moving forward, continuous research initiatives and international collaborations especially for prospective research are crucial for refining treatment protocols and developing evidence-based guidelines for management of this condition, with the ultimate aim of maximizing outcomes for the affected children.

## Supplementary Information

Below is the link to the electronic supplementary material.

Supplementary file1 (DOCX 17 kb)

## Data Availability

No datasets were generated or analysed during the current study.
