# Supplementary material for: A decade of managing pediatric major traumatic vascular injuries: insights from a referral center
Source: Pediatr Surg Int. 2024 Nov 13;40(1):306. doi: 10.1007/s00383-024-05887-7 (PMC11557644; doi:10.1007/s00383-024-05887-7)
Supplement: Supplementary file 1 — Supplementary file1 (DOCX 17 kb) [file 383_2024_5887_MOESM1_ESM.docx]

Appendix:

- **Supplementary Table 1.1** Risk factors for mortality (patients, n=30)
- **Supplementary Table 1.2** Risk factors for mortality (continued) (patients, n=30)
- **Supplementary Table 2** Correlation between mortality and location of injury (vessel injured, n=48)

**Appendix**

**Supplementary Table 1.1** Risk factors for mortality (patients, n=30)

| Parameter | Survived until discharge | n | Mean Rank | Sum of Ranks | P value |
| --- | --- | --- | --- | --- | --- |
| Age (in months) | Yes | 27 | 15.35 | 414.50 | 0.80 |
|  | No | 3 | 16.83 | 50.50 |  |
|  | Total | 30 |  |  |  |
| Weight (in kg) | Yes | 27 | 15.78 | 426.00 | 0.65 |
|  | No | 3 | 13.00 | 39.00 |  |
|  | Total | 30 |  |  |  |
| Hemoglobin on arrival (in g/dl) | Yes | 27 | 16.13 | 435.50 | 0.25 |
|  | No | 3 | 9.83 | 29.50 |  |
|  | Total | 30 |  |  |  |

*Mann Whitney U test

**Supplementary Table 1.2** Risk factors for mortality (continued) (patients, n=30)

| Parameter | | Survived until discharge | | P value |
| --- | --- | --- | --- | --- |
|  |  | Yes (n=27) | No (n=3) |  |
| Gender | Male | 21 | 3 | 1.00 |
|  | Female | 6 | 0 |  |
| Mechanism | Firearms | 12 | 1 | 0.79 |
|  | Sharp object | 4 | 0 |  |
|  | Dog bite | 1 | 0 |  |
|  | Road traffic accident | 9 | 2 |  |
|  | Fall from height | 1 | 0 |  |
| Presence of shock on arrival | Yes | 14 | 3 | 0.24 |
|  | No | 13 | 0 |  |
| Management | Expectant | 4 | 1 | 1.00 |
|  | Endovascular | 1 | 0 |  |
|  | Surgical | 22 | 3 |  |
| Time to intervention | 3 or less | 10 | 2 | 0.74 |
|  | 4 to 6 | 4 | 1 |  |
|  | 7 to 9 | 3 | 0 |  |
|  | 10 to 12 | 1 | 0 |  |
|  | more than 12 | 6 | 0 |  |

*Fisher’s Exact test

**Supplementary Table 2** Correlation between mortality and location of injury (vessels injured, n=48)

| Parameter | | Survived until discharge | | | | P value |
| --- | --- | --- | --- | --- | --- | --- |
|  |  | Yes (n=39) | | No (n=9) | |  |
|  |  | n | % | n | % |  |
| Anatomical locations of arterial injury | Neck | 3 | 12.5 | 0 | 0 | 0.25 |
|  | Torso | 2 | 8.3 | 2 | 40 |  |
|  | Upper extremities | 2 | 8.3 | 0 | 0 |  |
|  | Lower extremities | 17 | 70.9 | 3 | 60 |  |
| Anatomical locations of venous injury | Neck | 2 | 13.3 | 0 | 0 | 1.00 |
|  | Torso | 5 | 33.3 | 2 | 50 |  |
|  | Upper extremities | 0 | 0 | 0 | 0 |  |
|  | Lower extremities | 8 | 53.4 | 2 | 50 |  |

*Fisher’s Exact test
